# Metal Concentrations in the Liver and Stable Isotope Ratios of Carbon and Nitrogen in the Muscle of Silvertip Shark (*Carcharhinus albimarginatus*) Culled off Ishigaki Island, Japan: Changes with Growth

**DOI:** 10.1371/journal.pone.0147797

**Published:** 2016-02-09

**Authors:** Tetsuya Endo, Osamu Kimura, Chiho Ohta, Nobuyuki Koga, Yoshihisa Kato, Yukiko Fujii, Koichi Haraguchi

**Affiliations:** 1 School of Pharmaceutical Sciences, Health Sciences University of Hokkaido, 1757 Kanazawa, Ishikari-Tobetsu, Hokkaido 061–0293, Japan; 2 Faculty of Nutritional Sciences, Nakamura Gakuen University, 5-7-1 Befu, Jonan-Ku, Fukuoka 814–0198, Japan; 3 Kagawa School of Pharmaceutical Sciences, Tokushima Bunri University, 1314–1 Shido, Sanuki, Kagawa 769–2193, Japan; 4 Daiichi College of Pharmaceutical Sciences, 22–1 Tamagawa-Cho, Minami-Ku, Fukuoka 815–8511, Japan; Sonoma State University, UNITED STATES

## Abstract

We analyzed Hg, Cd, Zn, Cu and Fe concentrations in liver samples as well as the Hg concentration and stable isotope ratios of carbon and nitrogen (δ^13^C and δ^15^N) in muscle samples from silvertip sharks (*Carcharhinus albimarginatus)* in Japan. Muscular and hepatic Hg concentrations increased with increased body length. However, these increases were more prominent in the liver than in the muscle samples, and appeared to occur after maturation. Hepatic Zn and Cu concentrations decreased during the growth stage, and then increased concomitantly thereafter with increases in Cd burden. Hepatic Fe concentration from males increased proportionally with increases in body length, whereas no increase was observed in samples from females, probably due to the mother-to-embryo transfer of Fe. The δ^13^C values tended to decrease with increases in body length, whereas no decrease in the δ^15^N values was observed.

## Introduction

Marine predators, particularly long-lived marine mammal and shark species, are known to accumulate high levels of mercury (Hg) which are biomagnified via the food web. The hepatic Hg concentration in mature marine mammals is greater than the muscular Hg concentration [1–4] as the hepatic Hg concentration increases greatly after maturation due to the formation of Hg-Se complexes [3–6]. In contrast, the available data on the comparative Hg concentrations in the muscle and liver of shark species throughout their life spans are limited and somewhat inconsistent [7–10].

We have previously investigated the Hg concentrations in liver and muscle samples from three shark species at different stages during their life span, and identified different patterns in Hg distribution: the Hg concentration in the liver and muscle samples of star-spotted dogfish (*Mustelus manazo*) increased markedly after maturation [7], the Hg concentration in the liver samples of spiny dogfish (*Squalus acanthias*) increased slightly with increases in body length, while that in the muscle samples increased markedly after maturation [8], and the Hg concentration in the liver samples of tiger sharks (*Galeocerdo cuvier*) increased markedly after maturation, while that in the muscle samples increased in proportion to body length [9]. On the other hand, Branco et al. [10] analyzed the Hg concentrations in liver and muscle of blue sharks (*Prionace glauca*) and reported similar levels of Hg in the liver and muscle that increased slightly with increases in body length: they did not observe any marked increases in Hg due to the formation of Hg-Se complexes. de Pinho et al. [11] reported that the Hg concentrations in the muscle of five shark species increased with increases in their body lengths, but they did not determine the hepatic Hg concentrations.

In shark species, males are generally smaller than females and, on average, reach their maximum body length more quickly than females, whereas females tend to mature later and live longer than males [12]. The Hg concentration in the liver and/or muscle is generally higher in males than in females of similar body length, and the marked increase in Hg in the liver and/or muscle after maturation generally proceeds more quickly in males than in females due to the faster growth and earlier cessation of maturation in the males of species such as spiny dogfish [8,13], shortspine dogfish (*Squalus mitsukurii*, [14]), star-spotted dogfish [7] and several shark species [15]. In contrast to most shark species, the growth rates of male and female tiger sharks are reported to be similar [16], and the body lengths of males and females, reflected in the marked increases in Hg in the liver after the maturation, are also similar [9].

Compared with the star-spotted dogfish and tiger shark, the spiny dogfish has a relatively large, lipid-rich liver, and the Hg concentration in the liver is trace. We previously speculated that liver lipid content and liver size could affect Hg distribution, as Hg may be sparingly distributed in organs containing large amounts of lipids [7,17]. As part of our investigation of the factors affecting Hg distribution in shark species, we have analyzed the amount and composition of liver oils (color) as well as liver size [7,9] to clarify the above-mentioned three patterns of Hg accumulation in the liver and muscle observed among spiny dogfish, star-spotted dogfish and tiger sharks. However, we have not yet identified the factors affecting Hg distribution.

Cadmium (Cd) is accumulated in the liver of tiger sharks in a body length (age)-dependent manner [9]. On the other hand, essential metals, such as zinc (Zn) and copper (Cu), in the liver of tiger sharks decrease during the growth stage, and then increase with a concomitant increase in Cd burden after maturation. High concentrations of Zn and Cu during the growth stage are reported in the livers of mammals and fish [1,18–20], and post-maturation increases in Zn, Cu and Cd concentrations in the liver, which induce the synthesis of metallothionein (MT) in the liver and subsequently bind to it, are reported in marine mammals [20,21]. However, the changes in Zn, Cu and Cd concentrations throughout their life span have not yet been compared between male and female shark species with different growth rates. A well-designed large-scale survey is necessary to investigate the changes in Hg, Cd, Zn and Cu concentrations throughout the animals’ life span as well as to clarify the gender-related differences in the concentrations of those metals due to differences in growth rate.

We analyzed the stable isotope ratios of carbon and nitrogen, generating delta notation values (δ^13^C and δ^15^N), in muscle samples of three shark species in order to compare their ecology. The relationships between the δ^13^C or δ^15^N value and body length in spiny dogfish [8] and star-spotted dogfish [7] differed from those in tiger sharks [9]: both the δ^15^N and δ^13^C values in the spiny dogfish and star-spotted dogfish increased with increases in body length (growth), while those in tiger sharks decreased. The increases in the δ^15^N and δ^13^C values due to growth in the spiny dogfish and star-spotted dogfish may be explained by growth-related increases in their trophic levels [7,8], while the decreases in the δ^15^N and δ^13^C values with growth in the tiger shark may be explained by a shift from coastal to pelagic feeding [9]. Furthermore, the Hg concentrations in the liver of the spiny dogfish and star-spotted dogfish increased with increases in δ^15^N value, while that in the liver of tiger sharks did not show a simple increase: the Hg concentration in tiger shark appears to peak at a δ^15^N value between 11 and 12 ‰ and a δ^13^C value between -16.0 and -15.5 ‰. The reason for the possible peaks in Hg at these δ^15^N and δ^13^C ranges in tiger sharks remains to be clarified. Further study of the relationships among the δ^15^N and δ^13^C values and Hg concentration in shark species is necessary to confirm and explain the different patterns of Hg distribution found in the three shark species and the possible peaks in Hg concentration.

The silvertip shark (*Carcharhinus albimarginatus*) is a large shark with placental viviparous (yolk sac placenta) reproduction. According to Compagno et al. [22], this shark is 0.63–0.68 m (total length; TL) at birth and reaches a maximum body size of ~3.00 m TL. Males and females mature at about 1.60–1.80 m TL and 1.60–1.99 m TL, respectively, and the growth rates are very slow among shark species. The immature sharks inhabit shallow waters close to the shore, with the mature animals inhabiting a wider range. The mature sharks feed on a variety of midwater and bottom fishes, eagle rays and octopus. According to Last and Stevens [23], the growth of silvertip sharks is relatively slow (about 9 cm a year for juveniles) in comparison to other shark species, and they inhabit over or adjacent to continental or insular shelves, coral reefs and offshore banks. However, the life span and relationship between age and body length for male and female silvertip sharks have not yet been reported. We previously analysed Hg, Cd, Zn, Cu and Fe concentrations in liver and muscle samples from 8 immature silvertip sharks, and reported that Hg, Cd and Fe concentrations in muscle and liver did not increase with increases in body length, while Zn and Cu concentrations in the liver tended to decrease [24]. Further analyses of silvertip sharks, including a large number of mature animals and measurements of δ^13^C and δ^15^N, were necessary to clarify the body length (life stage)-related changes in Hg, Cd, Zn, Cu and Fe concentrations, the gender-related differences in the those metal changes, and the relationships among body length, δ^15^N and δ^13^C values.

The aim of the current study was to compare our previous findings for spiny dogfish, star-spotted dogfish and tiger sharks with those for silvertip sharks as well as to confirm our above mentioned speculations. To this end, we collected further muscle and liver samples from male and female silvertip sharks at various stages of their life span, and determined the Hg, Cd, Zn, Cu and Fe concentrations in the liver samples and the Hg concentration and δ^15^N and δ^13^C values in the muscle samples. Furthermore, we measured the amount of liver oil and liver size for comparison with those of the three other shark species mentioned above.

## Materials and Methods

### Ethics statement

The collection of liver and muscle samples from culled silvertip sharks **(***Carcharhinus albimarginatus*) for the purpose of this study was carried out with the permission of the Yaeyama Fishery Cooperative, Ishigaki Island. The Animal Research Ethics Committee of the Graduate School of Pharmaceutical Sciences, Health Sciences University of Hokkaido does not cover the study of dead fish.

### Sampling

Liver and muscle samples from the silvertip sharks were randomly collected during the culling of shark species undertaken in the summer of 2007 (n = 8), 2008 (n = 11), 2011 (n = 20), 2012 (n = 8), 2013 (n = 15) and 2014 (n = 19) off Ishigaki Island, Okinawa Prefecture, Japan, and were stored at -20°C until analysis. Samples were obtained from 32 male and 39 female sharks. Liver and muscle samples from two embryos were also obtained from pregnant sharks collected in 2011 and 2012, respectively. We measured the precaudal length (PCL) of all silvertip sharks collected and the total length (TL) of some sharks randomly chosen, to obtain the equation for describing the relationship between the PCL and TL for silvertip sharks.

### Chemical analyses

Total mercury (Hg) in the shark samples was determined using a flameless atomic absorption spectrophotometer (Hiranuma Sangyo Co. Ltd., HG-310) after digestion by a mixture of HNO_3_, HClO_4_ and H_2_SO_4_ [25]. Cadmium (Cd), zinc (Zn), copper (Cu) and iron (Fe) were determined using a Z-8100 Hitachi Polarized Zeeman flame and flameless atomic absorption spectrophotometer after digestion by HNO_3_ and HClO_4_ [9]. DORM-2 (National Research Council of Canada), a certified reference material for Hg (4.64 μg/g), Cd (0.043 μg/g), Zn (25.6 μg/g), Cu (2.34 μg/g) and Fe (142 μg/g), was used as an analytical quality control for the respective metals. The mean recoveries of Hg, Cd, Zn, Cu and Fe with S.D. were 95 ± 3, 78 ± 5, 98 ± 3, 87 ± 6 and 90 ± 7% (n = 3–5) of the certified values for DORM-2, respectively. All metal concentrations in the liver and muscle samples are presented on a wet weight basis.

The stable isotope ratios of carbon (δ^13^C) and nitrogen (δ^15^N) in the muscle and liver samples, after the removal of lipids by chloroform/methanol (2:1) extraction [26], were determined using a mass spectrometer (Delta S, Finnigan MAT, Bremen, Germany) coupled with an elemental analyzer (EA1108, Fisons, Roano, Milan, Italy) held in the Center for Ecological Research (CER), Kyoto University. As reported previously [27], we used CERKU-1, -2 and -5 [28] as the working reference materials for δ^13^C and δ^15^N. Ratios of C:N values in all samples analyzed were below 3.3.

Lipids (oil) in minced fresh liver samples were extracted three times using hexane. The combined extracts were concentrated and the HEL (hexane extractable lipid) content was determined gravimetrically [17].

### Statistical analyses

The data were analyzed by Student’s *t*-test and linear regression, using the Statcel 2 program. A value of P<0.05 was considered to be significant. All data are expressed as the mean ± standard deviation (S.D.). Curve fitting for Hg, Cd, Zn and Cu was undertaken using Excel (Mac 2011), and the inflection points and minimal values of these curves were calculated to estimate the gradual increasing points of Hg and Cd and the lowest points of Zn and Cu concentrations, respectively. Curve fitting of the high Hg, Cd, Zn, Cu and Fe concentrations to the normal curves without any statistical basis were undertaken to estimate the peaks for the distribution of those metals.

## Results

### Body length and body weight

Table 1 shows the body weight and length, the Hg concentration, δ^15^N and δ^13^C values in the muscle samples, and the Hg, Cd, Zn, Cu and Fe concentrations in the liver samples from male and female silvertip sharks, including the data for 8 silvertip sharks reported previously [24].

**Table 1:** Analytical results for male and female silvertip sharks (mean ±S.D.).

	Weight	Length	Muscle			Liver					
	(kg)	(PCL; m)	Hg (μg/wet g)	δ^15^N (‰)	δ^13^C (‰)	Hg (μg/wet g)	Cd (μg/wet g)	Zn (μg/wet g)	Cu (μg/wet g)	Fe (μg/wet g)	HEL (%)
Total	40.3	1.26	2.04	10.9	-16.2	3.34	1.26	8.54	1.71	31.12	38.1^a^
	±31.1	±0.32	±1.16	±0.41	±0.50	±6.56	±2.47	±4.62	±0.91	±20.59	±9.4
Range (n = 71)	6–147	0.65–1.90	0.58–5.80	10.0–12.0	-17.2 to -14.9	0.122–33.03	0.02–12.30	2.52–23.45	0.55–4.31	9.4–102	22.4–56.2
Male	37.6	1.27	2.24	10.9	-16.2	2.24	0.82	8.21	1.46	36.35	35.3^b^
(n = 32)	±21.7	±0.26	±1.14	±0.4	±0.5	±1.14	±1.41	±4.39	±0.37	±25.89	±8.1
Female	42.5	1.25	1.87	10.9	-16.3	3.71	1.61	8.82	1.92^#^	26.99^#^	40.5^c^
(n = 39)	±37.2	±0.36	±1.16	±0.4	±0.5	±7.96	±3.05	±4.83	±1.13	±13.88	±8.8

^#^Significantly different from males (P<0.05).

Numbers of samples measured HEL were 29^a^, 10^b^ and 19^c^, respectively.

**Fig 1:**
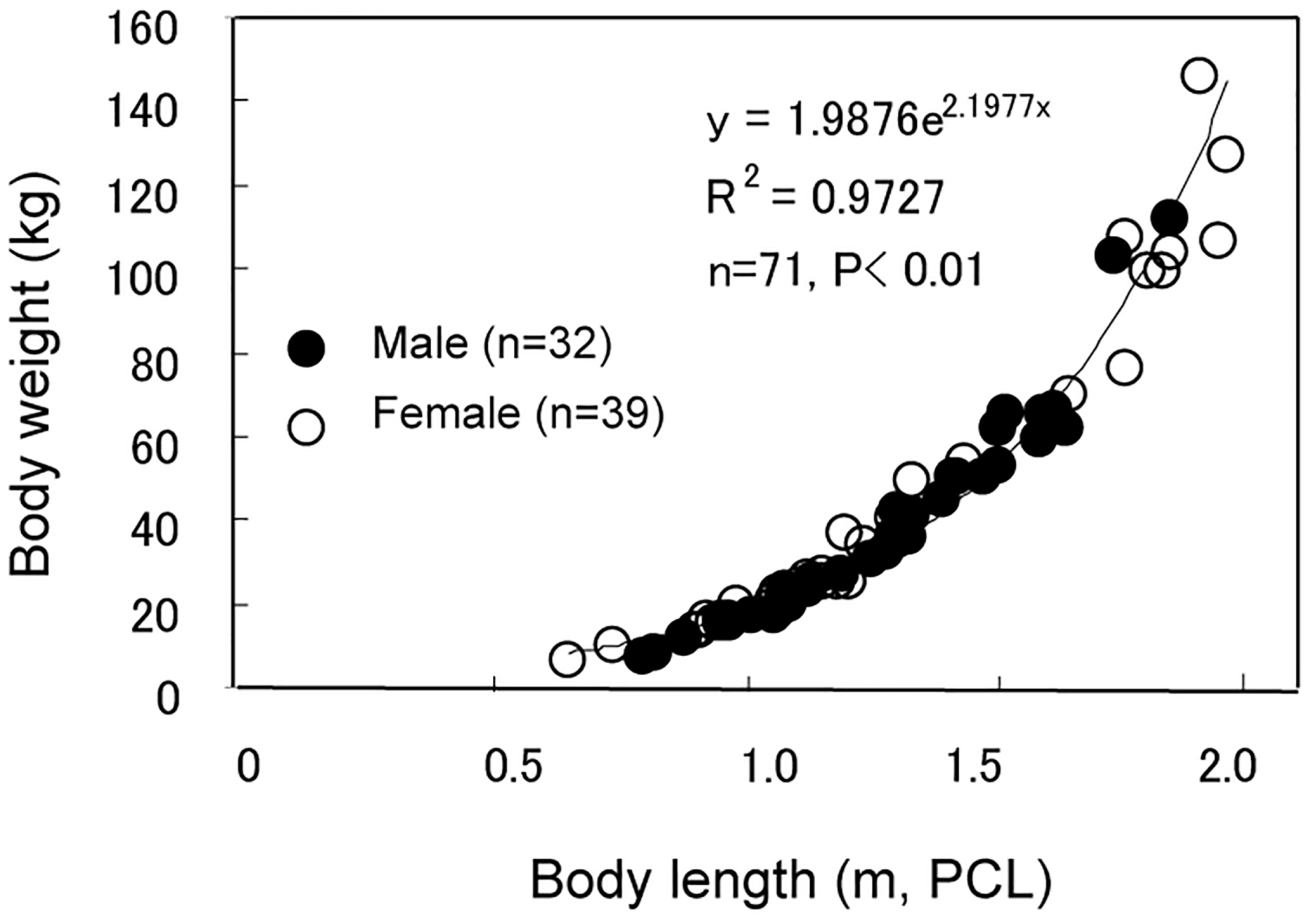
Relationship between length (PCL) and weight in male and female silvertip sharks.

The body length (PCL) and weight of male sharks determined (n = 32) were similar to those of the female sharks determined (n = 39) (P = 0.780 and P = 0.519, respectively; Table 1). The relationship between PCL and weight of the analyzed sharks is shown in Fig 1. The weight of the male and female sharks, as well as that of the combined male and female population, increased exponentially with increases in PCL (P<0.01). However, the size distributions of the male and female sharks shown in Fig 1 differed somewhat: among the 12 largest sharks exceeding 1.7 m PCL, only two were males. In addition, some of females larger than 1.7 m PCL were found to be pregnant.

The relationship between PCL and TL in the animals randomly chosen from the combined male and female silvertip shark population randomly chosen could be described as follows:

$$PCL(m)=0.7512\times TL(m)-0.0233\;({\text{R}}^{2}=0.997,\text{ n}=23,\text{ P}<0.01)$$

### Relationships between body length and non-essential metal (Hg and Cd) concentration

**Fig 2:**
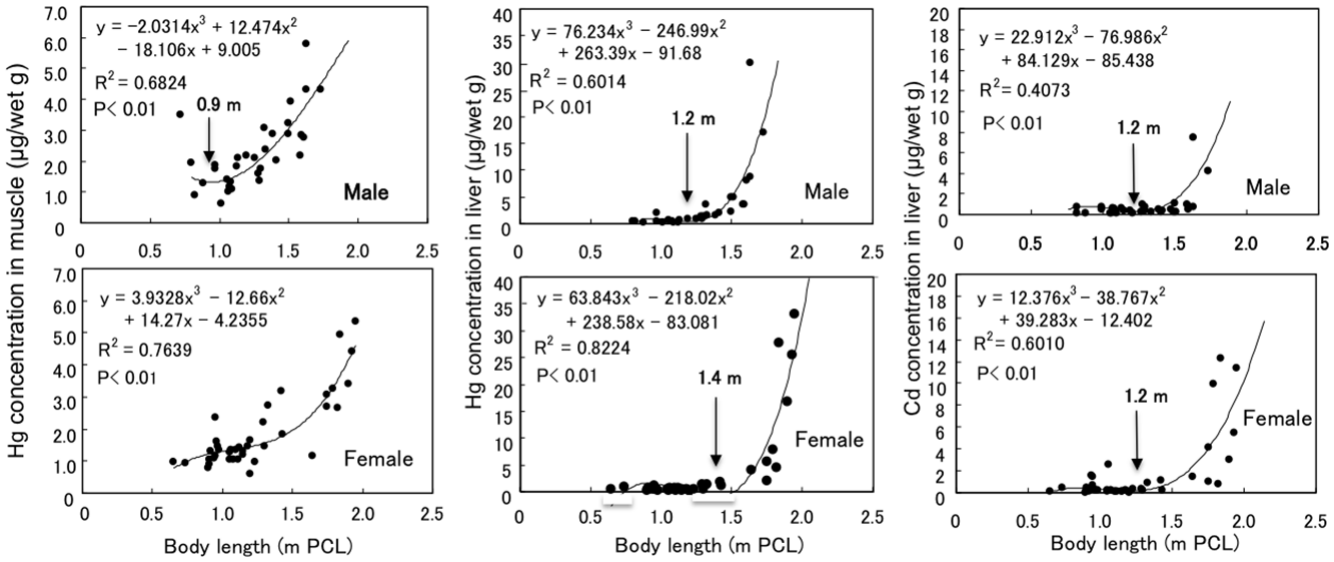
Relationships between length (PCL) and Hg or Cd concentration in the liver of male and female silvertip sharks.

The average Hg concentration in the muscle samples from males was similar to that for females (2.24 ± 1.14 μg/wet g, n = 32 vs 1.87 ± 1.16 μg/wet g, n = 39, P = 0.090) (Table 1). The average Hg concentration in the liver samples from males (2.24 ± 1.14 μg/wet g, n = 32) was similar to that for females (3.71 ± 7.96, n = 39 μg/wet g) (P = 0.860), although the three highest concentrations of Hg among the combined male and female samples (25.55, 27.72 and 32.03 μg/wet g) were observed in larger female sharks (see Fig 2).

The average Cd concentration in the liver samples from males (0.82 ± 4.39 μg/wet g, n = 32) was slightly lower than that for females (1.61 ± 3.05 g/wet g, n = 39) (P = 0.184) (Table 1), although the three highest concentrations of Cd among the combined samples (10.40, 11.38 and 12.30 μg/wet g) were observed in larger female sharks (see Fig 2).

The Hg concentrations in muscle and liver samples and the Cd concentrations in liver samples were plotted against body length (PCL), and curve fittings were undertaken to calculate the inflection points (Fig 2). The data can be adequately approximated by the cubic function (P<0.01) rather than the quadratic or linear function.

The Hg concentration in the liver and muscle samples from males (n = 32) and females (n = 39) increased with increases in length, although the increases in the liver samples were more prominent than those in the muscle samples, irrespective of gender. The Hg concentrations in the liver samples increased gradually in male and female sharks exceeding 1.2 m and 1.4 m PCL (inflection points), respectively, and markedly increased thereafter in males exceeding about 1.4 m and females exceeding 1.6 m PCL. On the other hand, the Hg concentrations in muscle samples increased gradually in males exceeding 0.9 m PCL; however, this point could not be calculated for females. The Hg concentrations in the liver samples were higher than those in respective muscle samples for all male sharks exceeding about 1.6 m PCL and female sharks exceeding about 1.7 m PCL. The Hg concentrations in the muscle and liver samples from males were both lower than those from females of the same body length.

The Hg concentrations in the muscle and liver samples from males and females (μg/wet g) were fitted to the following equations.

$$\text{Muscle for males: log}Hg=0.657\times PCL-0.527\;({\text{R}}^{2}=\;0.619,\text{ n}=32,\text{ P}<0.01)$$

$$\text{Muscle for females: log}Hg=0.520\times PCL-0.441\;({\text{R}}^{2}=\;0.648,\text{ n}=39,\text{ P}<0.01)$$

$$\text{Liver for males: log}Hg=1.901\times PCL-2.270\;(R^{2}=\;0.761\text{ n}=32,\text{ P}<0.01)$$

$$\text{Liver for females: log}Hg=1.508\times PCL-1.899\;({\text{R}}^{2}=\;0.721,\text{ n}=39,\text{ P}<0.01)$$

The average Cd concentration in males was similar to that in females (0.82 ± 0.1.41 μg/wet g, n = 32 vs 1.61 ± 3.05 μg/wet g, n = 39; P = 0.184, Table 1). The Cd concentration increased gradually in both male and female sharks exceeding 1.2 m PCL, and thereafter increased markedly in males exceeding about 1.4 m PCL and females exceeding about 1.6 m PCL (Fig 2). The Cd concentrations in the liver samples from males and females (μg/wet g) were fitted to the following equations.

$$\text{Liver for males: log}Cd=0.745\times PCL-1.261\;({\text{R}}^{2}=0.238,\text{ n}=32,\text{ P}<0.01)$$

$$\text{Liver for females: log}Cd=1.265\times PCL-1.909\;({\text{R}}^{2}=0.451,\text{ n}=39,\text{ P}<0.01)$$

### Relationships between body length and essential metal (Zn, Cu and Fe) concentration

The average Zn concentration in the liver samples from males (8.21 ± 4.39 μg/wet g, n = 32) was similar to that for females (8.82 ± 4.83 μg/wet g, n = 39) (P = 0.700), but the average Cu concentration for males (1.46 ± 0.37 μg/wet g, n = 32) was significantly lower than that for females (1.92 ± 1.13 μg/wet g, n = 39) (P<0.05) (Table 1).

**Fig 3:**
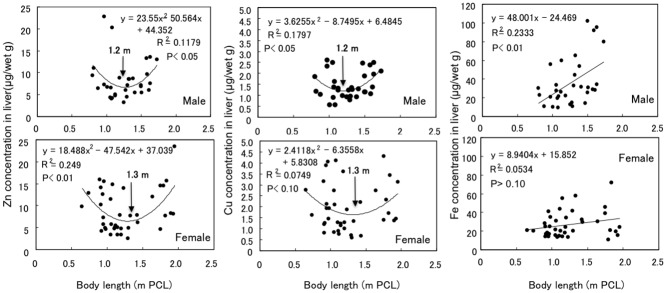
Relationships between length (PCL) and Zn, Cu or Fe concentration in the muscle of male and female silvertip sharks.

The curves for the Zn and Cu concentrations in the liver samples from males and females versus length (PCL) appear to be U-shaped (Fig 3): the Zn and Cu concentrations in the liver samples fitted to the quadratic functions rather than the linear or cubic functions, and the lengths at 1.2 m and 1.4 m corresponded to the lowest Zn and Cu concentrations for males and females, respectively.

**Fig 4:**
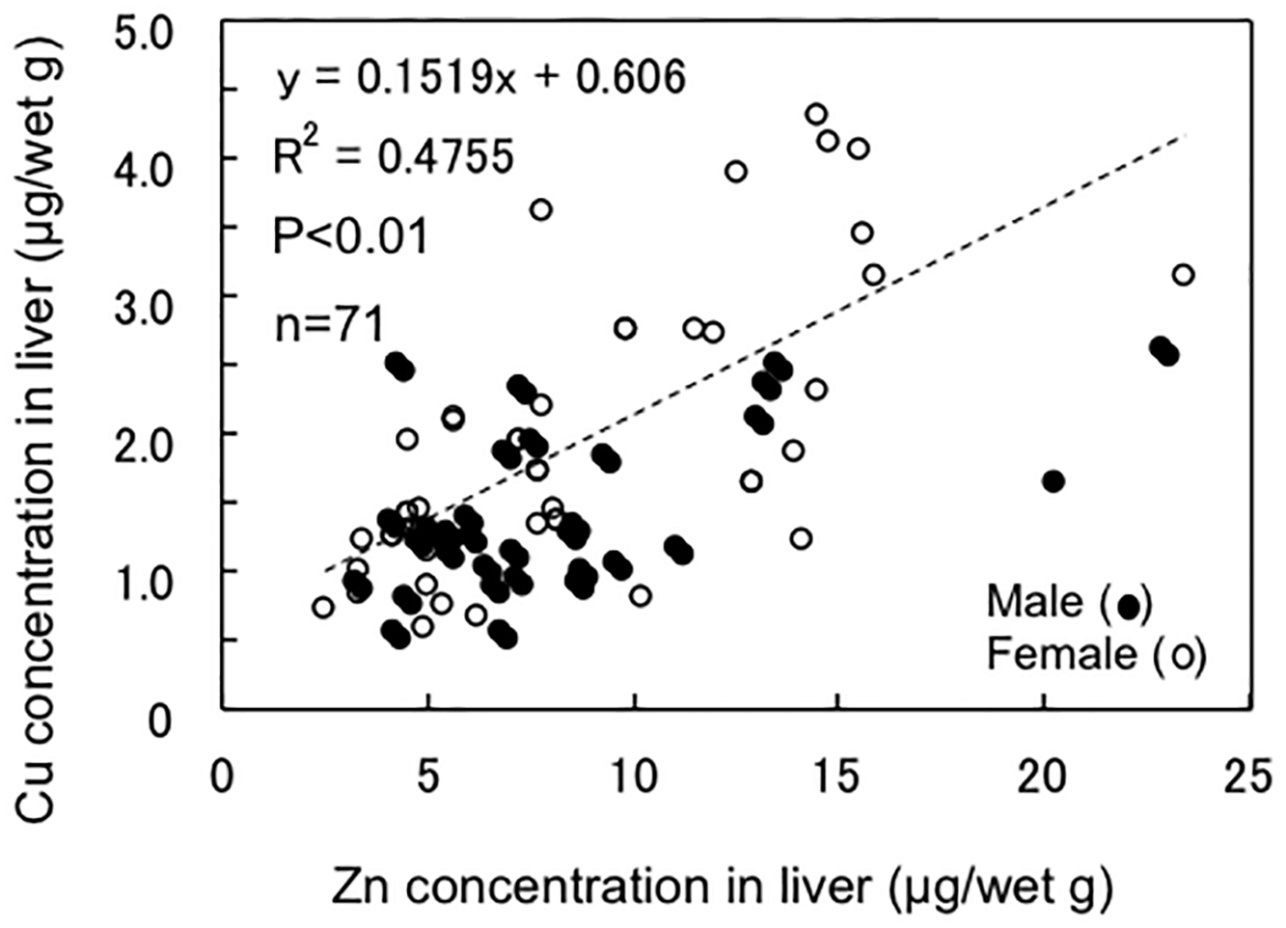
Relationship between Zn and Cu concentrations in the liver of silvertip sharks.

A significant correlation was found between the Zn and Cu concentrations in the combined liver samples from males and females (P<0.01, Fig 4). As data not shown in figures, significant positive correlations were also found between the Zn and Cd concentrations (R^2^ = 0.202, n = 71, P<0.01) and between the Cu and Cd concentrations (R^2^ = 0.108, n = 71, P<0.01), in spite of the U-shaped changes in Zn and Cu concentration (Fig 3).

The Fe concentration in the liver samples from males (36.35 ± 25.89 μg/wet g, n = 32) was significantly higher than that for females (26.99 ± 13.88 μg/wet g, n = 39) (P<0.05) (Table 1): the Fe concentration in males increased proportionally with increases in body length (P<0.01), while that in female did not increase (Fig 3).

### Relationships among body length and the stable isotope ratios (δ^13^C and δ^15^N)

The average δ^13^C and δ^15^N values for the male samples (n = 32) were similar to those for the female samples (n = 39) (Table 1), with average δ^13^C and δ^15^N values of -16.2 ± 0.51 ‰ and 10.9 ± 0.4 ‰ (n = 32), respectively, for the male samples and -16.3 ± 0.5 ‰ and 10.9 ± 0.4 ‰ (n = 39), respectively, for the female samples.

**Fig 5:**
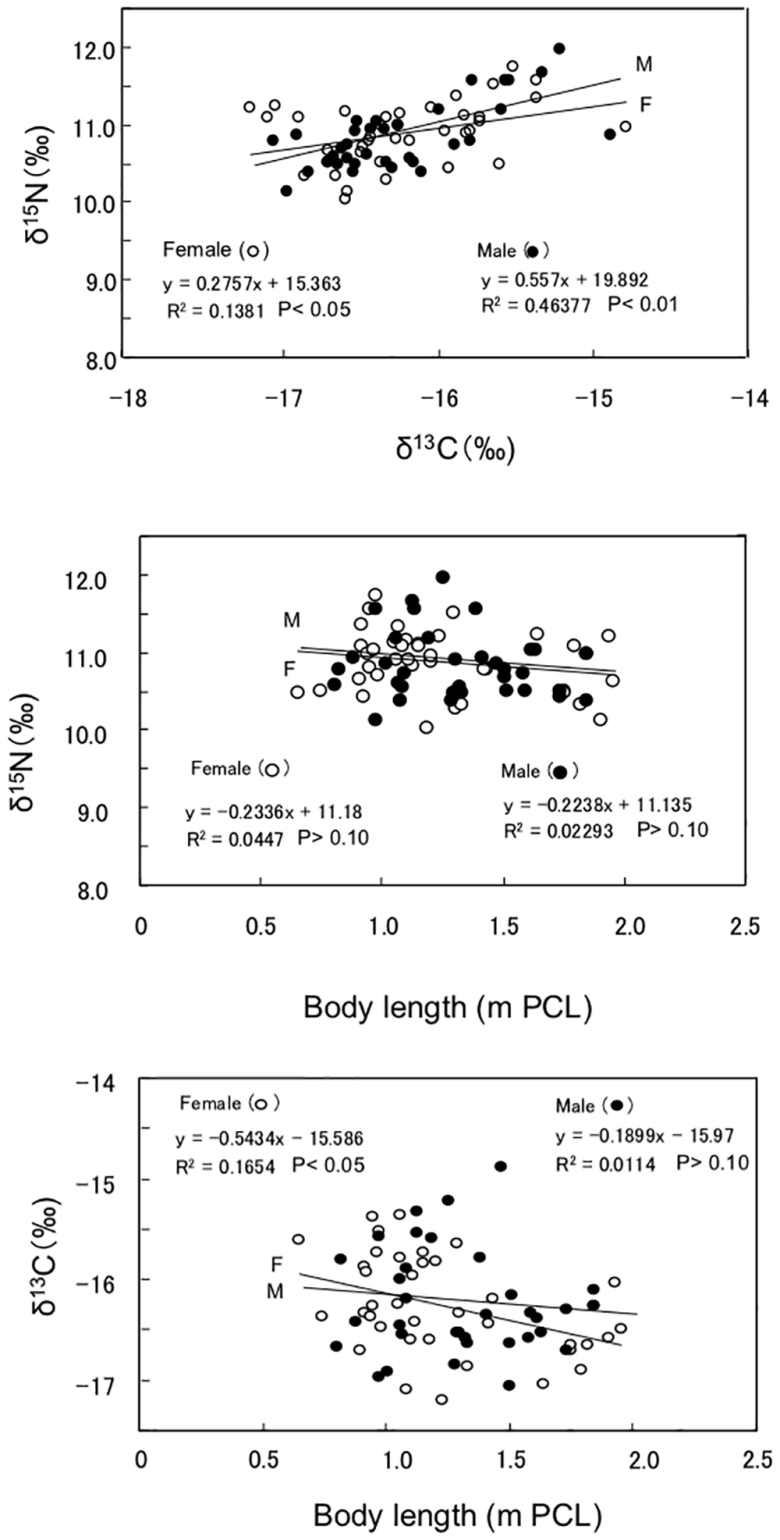
Relationship between Zn and Cu concentrations in the liver of silvertip sharks.

Fig 5 shows the relationships among the δ^13^C and δ^15^N values and body length. The δ^13^C values for the male and female samples correlated positively with their respective δ^15^N values (P<0.01 or 0.05), but the δ^15^N values for the male and female samples did not correlate with their body length (P>0.10). There was a significant negative correlation between the δ^13^C value for the female samples and their body length (P<0.05), whereas the correlation was not found for male samples (P>0.10).

### Relationships among stable isotope ratios and metal concentrations

**Fig 6:**
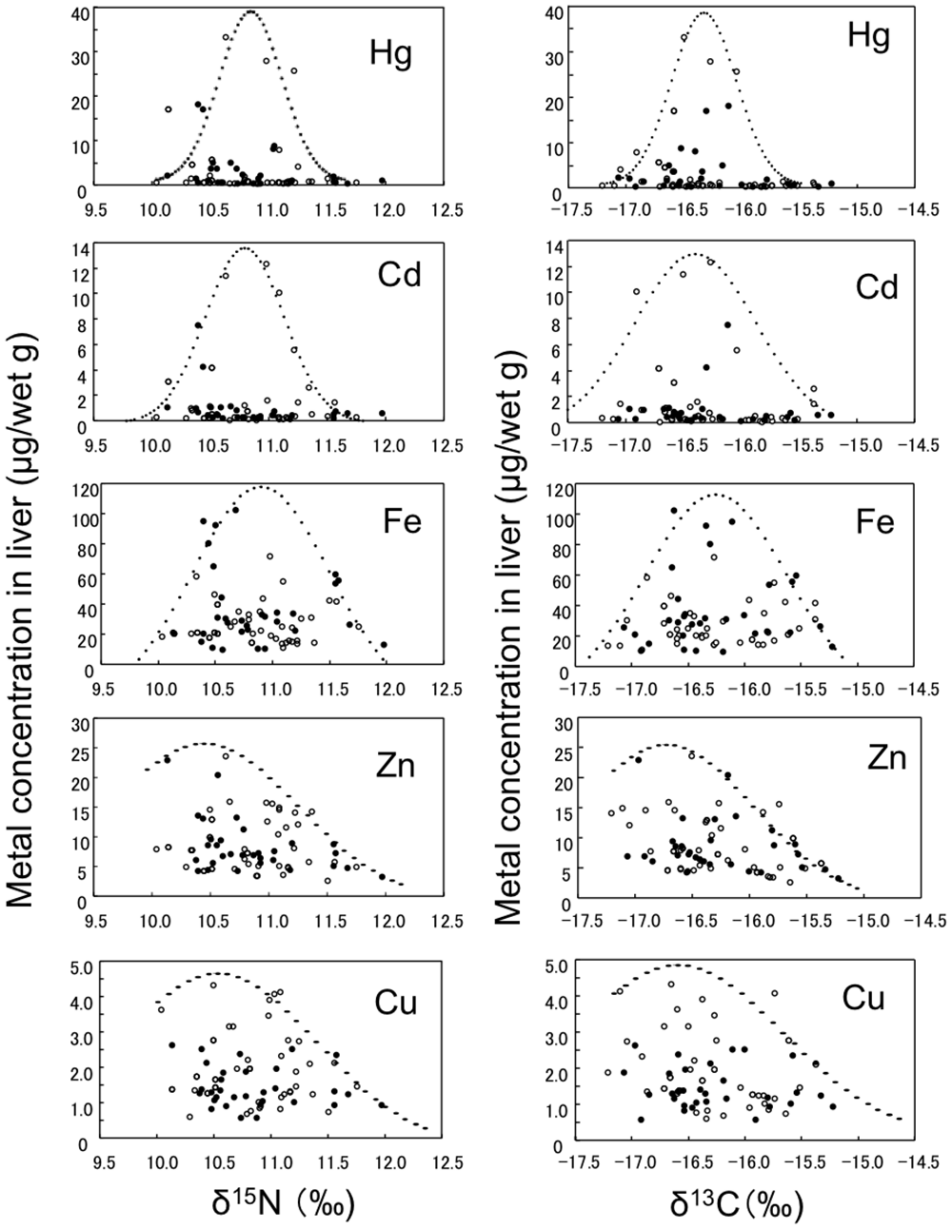
Relationships between δ^15^N or δ^13^C and metal concentrations in the liver of silvertip sharks Male (●), Female (○).

Fig 6 shows the metal concentrations in the liver plotted against the δ^15^N and δ^13^C values. Non-linear correlations were found between the metal concentrations and δ^15^N or δ^13^C value. From the supposed curves, the highest concentrations (peaks) of Hg, Cd and Fe were supposed to be at δ^15^N values between 10.5 and 11.0 ‰, and δ^13^C values between -16.5 and -16.0 ‰. In contrast, the peak Zn and Cu concentrations were unclear, but were speculated to exist at a δ^15^N value of approximately 10.5 ‰ and a δ^13^C value of approximately -16.5 ‰.

### Comparison of Hg concentration and stable isotope ratios between embryos and their mothers

The Hg concentrations in the muscle and liver samples and δ^13^C and δ^15^N values were compared between two embryo-mother pairs (0.40 m vs 1.90 m PCL and 0.45 m vs 1.75 m PCL) (Table 2).

**Table 2:** Analytical results for two silvertip shark embryo-mother pairs.

	Body length	Muscle			Liver		
	(m PCL)	Hg (μg/wet g)	δ^15^N (‰)	δ^13^C (‰)	Hg (μg/wet g)	δ^15^N (‰)	δ^13^C (‰)
Pair 1							
Embryo	0.40	0.924	10.6	-15.9	0.101	12.1	-15.1
Mother	1.90	3.410	9.9	-17.8	16.08	10.1	-16.9
Pair 2							
Embryo	0.45	1.160	13.0	-15.8	0.205	13.4	-14.3
Mother	1.75	3.080	10.5	-16.6	5.560	10.1	-16.3

The Hg concentrations in the muscle samples of from the two embryos (040 and 0.45 m PCL) were 0.924 and 1.160 g/wet g, respectively, and markedly lower than those of their mothers (3.410 and 3.080 g/wet g), respectively. The Hg concentrations in the liver samples from the two embryos were 0.101 and 0.205 μg/wet g, which were again markedly lower than those of their mothers (16.08 and 5.56 μg/wet g).

The δ^13^C and δ^15^N values in the muscle samples from the two embryos (0.40 and 0.45 m PCL) were -15.9 and 10.6 ‰, and -15.8 and 13.0 ‰, respectively, and both the δ^13^C and δ^15^N values were higher than those of the corresponding mothers (-17.8 and 9.9 ‰ and -16.6 and 10.5 ‰). Similar differences were found in the liver samples from the two embryo-mother pairs. All δ^13^C and δ^15^N values in the liver samples were higher than the corresponding values in the muscle samples.

### Relationships between body length and liver size or liver lipid content

We estimated the lipid content in the liver using HEL for randomly chosen male (n = 10) and female (n = 19) samples. The HEL for the male, female and combined samples were 35.3 ± 8.1%, 40.5 ± 8.8% and 38.6 ± 9.4%, respectively, and the combined HEL did not correlate with body length (R^2^ = 0.0636, n = 29, P>0.10).

We measured the hepatic weight of for sharks in randomly chosen male (n = 3) and female (n = 4) samples (the average of PCL was 1.73 ± 0.29 m, n = 7), and the data were expressed as the ratio of liver weight to body weight. The mean relative liver weight was 8.5 ± 2.1%, and this this was not correlated with body length (R^2^ = 0.1203, n = 7, P>0.10).

## Discussion

According to Compagno et al. [22], male and female silvertip sharks mature at about 1.60–1.80 cm TL and 1.60–1.99 cm TL, respectively, with a maximum body length of about 300 cm TL. These lengths correspond to PCLs of 1.2–1.4 m, 1.2–1.5 m and 2.25 m, respectively, based on the equation for the relationship between PCL and TL for the silvertip sharks analyzed in this study. Thus, the silvertip sharks analyzed in this study at smaller than 1.2 m PCL could be juvenile and immature animals, while the males exceeding 1.4 m PCL and females exceeding 1.5 m PCL could be mature animals.

The increases in Hg found in the liver samples from both the male and female silvertip sharks were more prominent than those in the respective muscle samples (Fig 2). The marked increases in Hg in the liver samples, probably due to the formation of Hg-Se complexes [3–6], are thought to occur in mature male and female sharks exceeding about 1.4 m and 1.6 m, respectively. For all mature silvertip sharks exceeding 1.6 m PCL for males and 1.7 m PCL for females, the Hg concentration was higher in the liver samples than in the respective muscle samples. As higher Hg concentrations were found in the liver samples than in the muscle samples in both mature star-spotted dogfish [7] and mature tiger sharks [9], this Hg distribution pattern may be a common indicator of maturation in shark species that form Hg-Se complexes in their livers. The determination of Se in their livers is, however, necessary to confirm this speculation.

As with the liver samples, the increases in Hg in the muscle samples are thought to occur faster in males than in females (Fig 2). Similar differences in the increases in Hg in the liver and muscle, attributable to the earlier cessation of growth in males than in females, were previously reported in the liver and/or muscle of spiny dogfish [8,13], shortspine dogfish [14], star-spotted dogfish [7] and a number of other shark species [15]. The marked increases in hepatic Hg occurred at body lengths of about 1.4 m and 1.6 m in male and female silvertip sharks, respectively (Fig 2). The difference in body length between male and female silvertip sharks was less than that in spiny dogfish [8] and star-spotted dogfish [7], but greater than that in tiger sharks [9]. The difference in growth rates between male and female silvertip sharks is probably smaller than those of spiny and star-spotted dogfishes and larger than that of tiger sharks.

Table 3 summarizes the analytical data of present study as well as our previous studies on spiny dogfish [8], star-spotted dogfish [7] and tiger sharks [9]. The average Hg concentration found in the muscle samples from the silvertip sharks (2.04 ± 1.16 μg/wet g, n = 71) is apparently higher than those in other shark samples such as spiny dogfish, shortspine dogfish, star-spotted dogfish and tiger sharks from Japanese waters (Table 3), as well as 11 shark species [15] and most demersal sharks [29] from Australian waters. The average Hg concentration found in the liver samples from the silvertip sharks (3.34 ± 6.56 μg/wet g, n = 71) is also apparently higher than that in spiny dogfish, star-spotted dogfish, and tiger sharks from Japanese waters (Table 3), blackmouth dogfish (*Galeus melastomus*) from the Mediterranean [30], blue sharks from Baja, California [31], and small-fin gulper sharks (*Centrophorus moluccensis*) from the western Indian Ocean [32]. The slow growth of silvertip sharks [22,23] may be one reason for the higher Hg concentrations in the liver and muscle. To explain the differences in Hg burden among shark species, biological data for silvertip sharks, such as growth rates, maximal body lengths and life spans for males and females, in addition to information on feeding preferences, are required. In agreement with our findings, Rumbold et al. [33] compared the relationship between Hg concentrations in the muscle and age in several shark species, and suggested that faster growing sharks display lower Hg concentrations.

**Table 3:** A comparison of analytical data for silvertip sharks with those of spiny dogfish, star-spotted dogfish and tiger sharks.

	Muscle (μg/wet g)	Liver (μg/wet g)	HEL (%)	Relative liver weight (%)
Spiny dogfish	0.354 ± 0.312 (n = 75) ^a^	0.027 ± 0.019 (n = 75) ^a^	63.5 ± 5.40 (n = 14) ^b^	13.6 ± 2.2 (n = 14) ^b^
male	0.316 ± 0.202 (n = 35) ^a^	0.023 ± 0.012 (n = 35) ^a^		
female	0.387 ± 0.378 (n = 40) ^a^	0.031 ± 0.022 (n = 40) ^a^		
Star-spotted dogfish	0.900 ± 0.527 (n = 61) ^b^	1.11 ± 1.30 (n = 61) ^b^	22.7 ± 3.7 (n = 14) ^b^	6.9 ± 2.7 (n = 14) ^b^
male	1.15 ± 0.57 (n = 20) ^b^	1.17 ± 1.73 (n = 20) ^b^		
female	0.776 ± 0.460 (n = 41) ^b^	0.607 ± 0.753 (n = 41) ^b^		
Tiger shark	0.86 ± 0.34 (n = 112) ^c^	1.32 ± 2.43 (n = 114) ^c^	38.1 ± 9.4 (n = 12) ^c^	17.1 ±1.7 (n = 16) ^c^
male	0.82 ± 0.32 (n = 50) ^c^	1.16 ± 2.87 (n = 52) ^c^		
female	0.89 ± 0.36 (n = 62) ^c^	1.46 ± 1.98 (n = 62) ^c^		
Silvertip shark	2.04 ± 1.16 (n = 71) ^d^	3.34 ± 6.56 (n = 71) ^d^	38.1 ± 9.4 (n = 10) ^d^	8.5 ± 2.1 (n = 7) ^d^
male	2.24 ± 1.14 (n = 32) ^d^	2.24 ± 1.14 (n = 32) ^d^		
female	1.87 ± 1.16 (n = 39) ^d^	3.71 ± 7.96 (n = 39) ^d^		

The data were quoted from Endo et al. (2009)[8]^a^, Endo et al. (2013)[10]^b^, Endo et al. (2015)[9]^c^ and the present study^d^.

HEL and relative live weight were determined from combined samples from male and female sharks.

The data were shown as mean ± S.D.

Marked increases in Hg, probably due to the formation of Hg-Se complexes with growth, were previously found in liver samples from blackmouth dogfish [30], star-spotted dogfish [7] and tiger sharks [9], but not in those from spiny dogfish [8]. We previously speculated that the formation of Hg-Se complexes hardly occur in larger livers with a high lipid content and also speculated that lipid composition could affect Hg-Se complex formation based on the fact that spiny dogfish have large livers with a high lipid content (HEL) and the color of the lipids extracted from spiny dogfish (transparent) differs from that from others species (amber). Our results showed that the HEL and relative liver weight of silvertip sharks (38.6 ± 9.4 and 8.5 ± 2.1%) were similar to those of star-spotted dogfish (22.7 ± 3.7 and 6.9 ± 2.7%), and apparently lower than those of spiny dogfish (63.5 ± 5.4 and 13.6 ± 2.2%) and tiger sharks (57 ± 8 and 17.1 ± 1.7%), respectively (Table 3). The distribution pattern of Hg in the muscle and liver of silvertip sharks (Fig 2) forms an intermediate pattern between that of star-spotted dogfish [7] and tiger sharks [9]. On the other hand, the distribution pattern of Hg found in spiny dogfish (i.e., the preferential accumulation of Hg in the muscle rather than in the liver) may be similar to that of shortspine dogfish [34], although the liver size and HEL of shortspine dogfish are unknown. Further determinations of Se and methylmercury are necessary to clarify the different patterns of Hg distribution among shark species.

Like Hg concentration, the hepatic Cd concentration increased markedly in male silvertip sharks exceeding about 1.4 m PCL and females exceeding about 1.6 m PCL, with gradual increases in Cd observed in males and females exceeding 1.2 m PCL (Fig 2). As mentioned above, the PCLs of 1.2 m, 1.4 m and 1.5 m are speculated to be the standard lengths of immature, mature male and mature female sharks, respectively. To our knowledge, this is the first report of gender-related differences in Cd accumulation attributable to differences in growth among shark species. The average Cd concentration in the liver samples was 1.26 ± 2.47 μg/wet g (0.02–12.30 μg/wet g, n = 71). This Cd level in the liver samples from silvertip sharks was higher than those observed in tiger sharks (0.09–7.59 μg/wet g; [9]) and ten shark species collected from British and Atlantic waters (0.09–2.6 μg/wet g: [35]). Silvertip sharks may preferentially eat Cd-contaminated species such as cephalopods and crustaceans [36]; however, this relatively high Cd concentration may be explained, not only by feeding preference, but also by the slower growth rate of silvertip sharks.

The regression lines for the Cd concentrations in the liver samples versus body length (PCL) of silvertip sharks were calculated as log*Cd* = 0.745×*PCL*−1.261 for males and log*Cd* = 1.265×*PLC*−1.909 for females. The slope of the regression line for Cd in the liver samples as well as the slopes for Hg in the muscle and liver samples for males (0.745, 0.657 and 1.901, respectively) and females (1.265, 0.520 and 1.508, respectively) were steeper than those for faster growing samples such as the tiger shark (the slopes for combined male and female samples were 0.317, 0.177 and 0.812, respectively; [9]). Furthermore, the slopes for Hg in the muscle and liver samples from male and female silvertip sharks were steeper than the respective slopes for muscle and liver samples from male and female star-spotted dogfish [7], and those for muscle samples from male and female spiny dogfish [8]. The steeper slopes for Cd and Hg found in silvertip sharks may be ascribed to their relatively slow growth rates and feeding preferences [22,23].

The Zn and Cu concentrations in the liver of smaller sharks decreased markedly with increases in body length until about 1.2 m PCL for males and about 1.3 m PCL for females, after which it was observed to increase (Fig 2). As mentioned above, these PCLs appear to be the cut-off points for differentiating between immature and mature male and female sharks. The more rapid decreases in Zn and Cu concentrations in males may be due to their faster maturation as essential metals such as Zn and Cu may be required at higher concentrations in newborn and rapidly growing animals, leading to decreases in concentration during the growth period [9]. We previously found decreases in Zn and Cu concentrations in the liver during the growth stage in tiger sharks, but did not find any gender-related differences in Zn or Cu concentration in tiger sharks as there is little difference in growth rate between males and females [16]. Further analyses of Zn and Cu concentrations in the liver of male and female star-spotted dogfish, the growth rates of which differ markedly, are necessary to confirm the more rapid decreases in Zn and Cu concentrations in males during the growth stage.

The Zn and Cu concentrations increased after maturation in both male and female silvertip sharks, and those increases appear to be correlated with similar increases in Cd (Figs 2 and 3). The ratio of Zn/Cu calculated from the regression lines in Fig 4 was about 6.6, and similar ratios of Zn/Cu were found in the livers of blue sharks (5.4) [31], Greenland sharks (*Somniosus microcephalus*) (3.9) [37], and tiger sharks (4.4) [9]. Significant positive correlations were also found between the Zn and Cd concentrations (P<0.01) and between the Cu and Cd concentrations (P<0.01) in the silvertip sharks, in spite of the U-shaped changes in Zn and Cu concentrations throughout the sharks’ life span (Fig 3) and the marked increase in Cd after maturation (Fig 2).

The average concentrations of Zn, Cu and Cd in the liver samples from the silvertip sharks were 8.51 ± 4.62, 1.26 ± 2.47 and 1.73 ± 0.93 μg/wet g, respectively (Table 1), and those for tiger sharks were 4.47 ± 3.72, 1.87 ± 1.04 and 0.34 ± 0.76 μg/wet g, respectively [9], with the Zn and Cd concentrations in the liver samples in silvertip sharks higher than those in the tiger sharks. A higher Cd burden could induce greater levels of MT synthesis in the liver of silvertip sharks, and the majority of the Cd could bind to MT along with Zn and Cu.

The Fe concentrations in the liver samples from the silvertip sharks differed significantly between males and females as the Fe concentration in the males increased proportionally with increases in body length, while that in females did not increase (Fig 3). As reproduction in silvertip sharks is placental viviparous, a certain proportion of the Fe may be transferred from the mother to the embryo. Taguchi et al. [14] suggested that the Fe concentration in the muscle of the embryo was higher than that in the mature shortspine dogfish. Interestingly, Honda et al. [38] reported that the Fe concentration in the liver of the male southern minke whale increased with age, whereas that in the female whale tended to decrease with age after about 10 years: the Fe concentration was the highest in resting females and decreased as gestation progressed. We first reported gender-related differences in Fe concentration in the liver of shark species, probably due to the mother-to-embryo transfer of Fe. A similar level of Fe to that found in silvertip sharks (31.1 ± 20.6 μg/wet g) was reported in the liver of five shark species from the eastern Mediterranean Sea (35–80 μg/wet g) [39], while an even higher level was reported in the liver of blue sharks (195.67 ± 95.57 μg/wet g, n = 35) [31].

As Hg is a typical contaminant that accumulates via the food web, positive correlations between the δ^15^N value and Hg concentration (burden) have been reported in many marine fish and mammals [40,41]. In agreement with this, we previously reported positive correlations between the δ^15^N value and Hg concentration in the liver and/or muscle samples from spiny dogfish [8] and star-spotted dogfish [7]. In this study, however, we did not find a positive correlation between the δ^15^N value in the muscle samples and Hg concentration in the liver samples from silvertip sharks (Fig 6). We speculated that the peak Hg concentration as well as the peaks for Cd and Fe in liver samples existed at a δ^15^N value of between 10.5 and 11.0 ‰ and at a δ^13^C value of between -16.5 and -16.0 ‰. Similar, but not-so-obvious peaks for Hg and Cd were found at similar δ^15^N and δ^13^C values in liver samples from tiger sharks [9]. The reason for the peak Hg and Cd concentrations observed in silvertip sharks and tiger shark remains unclear, but the higher concentrations of Hg and Cd in the silvertip sharks than in the tiger sharks may contribute to the prominent estimated peaks for Hg and Cd in the liver of silvertip sharks.

The estimated peaks for Zn and Cu were found at δ^15^N and δ^13^C values similar to those for the Hg, Cd and Fe peaks (Fig 6). However, these peaks were less prominent than those for Hg, Cd, and Fe, probably due to the U-shaped changes in Zn and Cu (Fig 3). In contrast, the estimated peaks for Zn and Cu observed in silvertip sharks were not found in tiger sharks [9]. Possible reasons for this difference between silvertip sharks and tiger sharks are (1) the Zn concentration is higher in silvertip sharks than in tiger sharks, although the Cu concentration is similar, and (2) the growth rate of tiger sharks [42] could be faster than that of silvertip sharks [22,23].

The δ^13^C value in the liver samples from female silvertip sharks decreased significantly with increases in body length (P<0.05), whereas that for males tended to decrease (P>0.05) (Fig 5). However, no body length-related changes were observed in the δ^15^N values for silvertip sharks. The changes in the δ^13^C and δ^15^N values found in male and female sharks due to increases in body length (Fig 5) may reflect the variety of prey items [22] and relatively localized movement [23]. We previously reported that the δ^13^C and δ^15^N values in the combined liver samples from male and female tiger sharks decreased significantly with increases in body length [9], while the δ^13^C and δ^15^N values in the liver samples from spiny dogfish and star-spotted dogfish increased significantly with increases in body length [7,8]. Further study is necessary to explain the reason for the contradictory changes in δ^13^C and δ^15^N among these four shark species. It is noteworthy that the peak Hg concentration in the scalp hair of donors from seven countries was estimated to coincide with δ^13^C values between -19 and -18 ‰, which appears to reflect their consumption of many food items with different δ^13^C values [43].

The δ^15^N and δ^13^C values in the muscle as well as the liver samples from silvertip sharks were higher in the embryo than in the mother, and the Hg concentrations were lower in the embryo than in the mother (Table 2), suggesting the preferential transfer of nutrients and the restricted transfer of Hg from the mother to the embryo. Silvertip sharks reproduce by placental viviparity [22], and higher δ^15^N values in the embryo than in the mother are reported in other shark species with viviparous reproduction [37,44]. In contrast, the Hg concentrations in the embryos are reported to be markedly lower than those in pregnant sharks [45], regardless of whether the reproduction modes of the sharks; i.e., placental viviparity or aplacental viviparity [22]. We previously compared the δ^13^C and δ^15^N values and the Hg concentrations between a embryo-mother pair of tiger sharks, which are known to reproduce by aplacental viviparity [46], and reported higher δ^13^C and δ^15^N values and the lower Hg concentration in the embryo [9]. In disagreement with our results, Bourg et al. [32] reported lower δ^15^N values in the embryos of deep-sea sharks with aplacental viviparous reproduction. Taguchi et al. [14] compared the concentrations of Hg, Cd, Zn, Cu and Fe in muscle samples from embryos and mature shortspine dogfish, which reproduce ovoviviparously [22], and reported higher concentrations of Cd, Zn, Cu and Fe in the embryos. Further comparisons of the δ^13^C, δ^15^N and Hg concentrations, in addition to those of Fe and other essential metals, in embryo-mother pairs of other shark species with different modes of reproduction are needed to clarify the mother-to-embryo transport of metals and nutrients in shark species. The δ^15^N and δ^13^C values in the silvertip shark embryos and mothers were higher in the liver than in the muscle (Table 2). The isotopic half-life of the liver is generally shorter than that of their muscle [47], and the δ^15^N and δ^13^C values of mature shark species are usually higher in the liver than in the muscle (our unpublished data).

In conclusion, the Hg concentrations in the muscle and liver samples from male silvertip sharks were higher than those in the samples from female sharks of similar body length, reflecting the slower growth rate and faster cessation of growth of the males. Similarly, the Hg and Cd concentrations in liver samples increased markedly in male silvertip sharks exceeding about 1.4 m PCL and female silvertip sharks exceeding about 1.6 m PCL, followed by the gradual increases in both metals. The Zn and Cu concentrations in the liver samples decreased during the growth stage, probably reflecting decreases in the physical requirements for Zn and Cu, and increased thereafter concomitantly with increases in Cd and the induction of MT. The Fe concentration in the liver samples from males increased proportionally with increases in body length, while that for females did not increase after maturation, probably due to the mother-to-embryo transfer of Fe. The δ^13^C values tended to decrease with increases in body length, whereas the δ^15^N values did not increase. The Hg and Cd concentrations tended to be highest at a δ^15^N value between 10.5 and 11.0 ‰ and a δ^13^C value between -16.5 and -16.0 ‰, probably reflecting feeding preferences and inhabiting area.
